# The Mildmay Mission Hospital

**Published:** 1893-02-25

**Authors:** William Gauld

**Affiliations:** Superintendent


### EDITOR'S LETTER-BOX.

[Our correspondents are reminded that prolixity is a great bar to publication, and that brevity of style and conciseness of statement greatly facilitate early insertion.]

### THE MILDMAY MISSION HOSPITAL.

SIR,—We have to thank you for the kind notice of this hospital in your issue of February 4th. May I beg the favour of your inserting a brief explanation of two points on which almost the only unfavourable criticism is made. The first is the number of cots in the children's ward as compared with the beds in the other wards for adults. The number of cots is put down at 20. This is a mistake, as there are only 18 in the large ward, to 14 beds in each of the other wards. Not a very great disproportion, we think. The second refers to the operating room, which is not quite finished yet. When the walls are ready for it, we intend to have them painted with enamel paint above the tiled dado, so as to be readily cleansed. Or, if the funds admit of it, we may have the whole tiled.

WILLIAM GAULD, M.D., Superintendent.

*** We regret the unintentional error in the number of beds in the children's ward; but our main point, which is that the theory that children require less cubic space than adults is erroneous, is not touched. With regard to the operation-room, our remarks referred not so much to the wall surfaces as to the numerous avoidable angles and recesses to be found in the sashes and doors, &c.—ED.

